# Breeding Habitat Distribution of Medically Important Mosquitoes in Kurunegala, Gampaha, Kegalle, and Kandy Districts of Sri Lanka and Potential Risk for Disease Transmission: A Cross-Sectional Study

**DOI:** 10.1155/2020/7915035

**Published:** 2020-09-02

**Authors:** Koshila Ranasinghe, Nayana Gunathilaka, Deepika Amarasinghe, Lahiru Udayanga

**Affiliations:** ^1^Department of Zoology and Environmental Management, Faculty of Science, University of Kelaniya, Ragama, Sri Lanka; ^2^Department of Parasitology, Faculty of Medicine, University of Kelaniya, Ragama, Sri Lanka; ^3^Department of Biosystems Engineering, Faculty of Agriculture & Plantation Management, Wayamba University of Sri Lanka, Makadura, Sri Lanka

## Abstract

Some arbovirus infections, especially dengue, have increased rapidly over the last few decades in Sri Lanka. Prevalence and distribution of different mosquito species have been limitedly documented, which remains grossly inadequate in providing evidence for potential health risks. In this study, the diversity and species composition of mosquitoes in four selected districts in Sri Lanka (Kurunegala, Gampaha, Kegalle, and Kandy) were investigated. Entomological surveys were conducted from a total of 160 temporary and permanent mosquito breeding habitats identified in the study area from June 2017 to October 2018. Mosquito immature stages were sampled using standard dipping, siphoning, or pipetting methods and identified up to the species level. Percentage relative abundance and habitat characteristics such as species richness, dominance, and Shannon–Weiner diversity were calculated for each surveyed habitat type. Associations between co-occurring species were estimated by Hulbert's coefficient of interspecific association (C8). A total of 4663 mosquito larvae belonging to seven genera and fifteen species of mosquitoes were collected. The relative distribution of mosquito species differed significantly among the four studied districts (*X*^2^ = 143.248; *df* = 33; *P* < 0.001). According to Kruskal–Wallis statistics (*P* < 0.05 at 95% of significance), all diversity indices for immature stages of medically important mosquitoes varied significantly across different breeding sites. Paddy fields had the significantly highest species richness of 4.0 ± 2.82. The coefficients of interspecific association among all the recorded medically important vector mosquitoes were found negative during the present study. The findings of the current study would be useful to identify the entomological potential for disease transmission and facilitate the implementation of appropriate vector control interventions. This would ultimately provide an avenue to improve the personal skills of health staff rather than limiting their knowledge to specified disease vectors, under which the control program is concerned.

## 1. Introduction

Vector-borne diseases have emerged as a serious public health concern, especially in tropical countries including Sri Lanka. Not surprisingly, arboviruses have caused vast epidemics with impressive numbers of patients or deaths. Sri Lanka has been suffering from mosquito-borne diseases since ancient times with the high prevalence of malaria, filariasis, and Japanese encephalitis (JE) [1]. A total of 159 species of mosquitoes under 19 genera have been recorded in Sri Lanka [2].

At present, dengue has grown up to a regular endemic and an important public health problem in Sri Lanka with over 40,000 cases reported annually [3]. Growing populations of vector mosquitoes due to unplanned urbanization, industrialization, and excessive population growth coupled with rural to urban migration have been identified as the major reasons for elevated dengue incidence in many countries including Sri Lanka [4]. Many mosquito species tend to select both natural and artificial containers as their breeding places [5]. They exploit almost all types of lentic aquatic habitats for their breeding [6].On the other hand, rice fields and marshy land habitats in Sri Lanka have significantly influenced the distribution of mosquito populations, including vector mosquitoes, thereby facilitating disease transmission [7]. Mainly larvae of *Culex* and *Anopheles* species are found in rice fields, nursery paddy beds, and large stagnant water bodies in Sri Lanka [8]. Ecological factors and physicochemical properties of water in the breeding habitat significantly affect the mosquito density and abundance [9]. The size, type, and nature of water body are found to be influensive in selecting the oviposition sites by mosquitoes [10].

Controlling immature stages of vector mosquitoes in their aquatic habitats is considered as the most effective mosquito controlling strategy [11]. Although vector control strategies have traditionally focused on killing mosquitoes using a variety of synthetic chemical insecticides, the development of insecticide resistance has declined the efficiency of killing mosquitoes. Also, the financial burden of insecticide-based vector control programs is a limiting factor for appropriate usage of larvicides and adulticides in many countries where mosquito-borne diseases remain endemic [12]. Therefore, an investigation of the prevalence and distribution of these mosquito immature stages may facilitate a better understanding of the distribution of different mosquito species.

Knowledge on where mosquitoes breed, their habitat preference over others, and the larval distribution are important parameters for risk assessment and implementation of sound mosquito control strategies. At present, dengue vectors have received a wider attention in Sri Lanka due to the rapid increase of dengue incidence [13]. However, there is a scarcity and scattered information about the mosquito fauna recorded from breeding habitats. The prevalence of different mosquito species from surveillance has been limitedly documented in Sri Lanka in recent years, which is grossly inadequate in providing evidence for potential health risks. The present investigation attempts to cater to this knowledge gap by documenting the prevalence of mosquito species in four selected districts of Sri Lanka.

## 2. Methods

### 2.1. Study Area

Four districts of Sri Lanka, namely Gampaha (Western Province), Kandy (Central province), Kegalle (Sabaragamuwa Province), and Kurunegala (Northwestern Province), representing climatically different regions, were selected for the study. The average climatological parameters of the study areas are indicated in Table 1.

### 2.2. Selection of Breeding Habitats and Entomological Investigations

**Figure 1:**
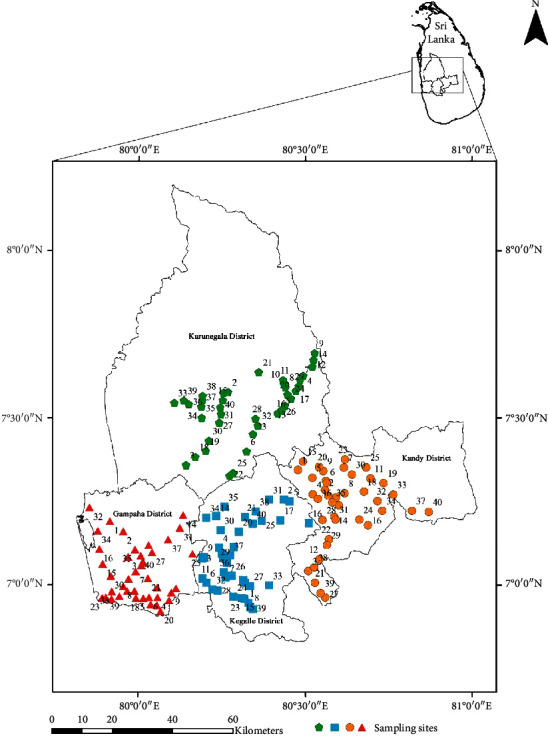
Sampling locations from the selected four district study sites: Kurunegala, Gampaha, Kegalle, and Kandy, Sri Lanka.

A preliminary survey was conducted from April to May 2017 to select districts and all possible sites conducive for mosquito breeding. A checklist of breeding habitats was prepared for all the study areas, including both permanent and temporary breeding habitats. Entomological surveys were conducted on a monthly basis from June 2017 to October 2018 at identified sampling locations (Figure 1), using standard dipping, siphoning, and pipetting methods according to the nature of the breeding habitat [14].

The location of the temporary or permanent breeding habitats was recorded using a portable global positioning (GARMIN eTrex SUMMIT) receiver. Collected mosquito larvae from 160 sampling sites were safely transported to the laboratory at the Department of Zoology and Environmental Management, University of Kelaniya, Sri Lanka.

### 2.3. Identification of Field-Caught Specimens

The field-caught larvae were first separated to the genus level and classified into instar stages (I, II, III, and IV). Larval stages III and IV were used for species identification directly. Other stages were reared at the insectary until reach to the stage III for species identification. Pupae were reared under insectary conditions, and adults emerged were processed for identification. Morphological keys were used to confirm the species identification [15–18].

### 2.4. Data Interpretation and Statistical Analysis

The percentage relative abundance of each species of mosquito immature stages was calculated. Habitat characteristics such as species richness, dominance, and Shannon–Weiner diversity were calculated for each habitat type. Distance-based redundancy analysis (dbRDA) was used to identify spatial similarities in mosquito community assemblages at the district level. Associations between co-occurring species were estimated by Hulbert's coefficient of interspecific association (C8) using presence-absence data as recommended by Hurlbert, 1969 [19]. Values for C8 could range from −1 to +1 for negative and positive associations, respectively. The formula used for calculating C8 is as follows:

(1) $$\begin{array}{c} \text{C}8=\frac{\text{ad}-\text{bc}}{\left|\text{ad}-\text{bc}\right|}\sqrt{\frac{{\text{Obs}}_{x^{2}}-{\text{Min}}_{x^{2}}}{{\text{Max}}_{x^{2}}-{\text{Min}}_{x^{2}}},} \end{array}$$

where a, b, c, and d are the values in four cells of a 2 × 2 contingency table; Obs *χ*^2^ is referred to the value of *χ*^2^ associated with the observed values of a, b, c, and d; Max *χ*^2^ is referred to the value of *χ*^2^ when a is as large (if ad ≥ bc) or as small (if ad *<* bc) as the marginal totals of the 2 × 2 table permit; and Min *χ*^2^ is referred to the value of *χ*^2^ when the observed a differs from its expected value (^a) by less than 1.00.

The chi-squared test of independence was used to evaluate the significance in the distribution of different mosquito species among different breeding sites and districts. The calculated diversity indices were subjected to the Kruskal–Wallis test followed by Dunn's multiple comparison (with Bonferroni adjustment) to identify the significance in the variations of diversity indices across breeding sites and the districts.

## 3. Results

### 3.1. Species Composition

A total of 4663 mosquito larvae belonging to fifteen species under seven genera were collected from natural and artificial water-holding macro- and microhabitats located in study sites during the sampling period (Table 2). The highest number of mosquito immature stages (*n* = 1554) were collected from Kurunegala district. *Culex *species were dominant in all the four districts followed by *Aedes* mosquitoes. *Culex gelidus* (26.45%) was the most predominant mosquito species in Kurunegala followed by *Cx. tritaeniorynchus* (25.35%). *Aedes albopictus* was the most abundant species in Gampaha and Kandy districts, accounting for 26.39% and 36.65%, respectively. Meanwhile, *Ae. aegypti* (19.19%) dominated in Kegalle district (Supplementary Annexure 1). Statistics of the chi-squared test of independence (*X*^2^ = 117.904; *df* = 3; *P* < 0.001) indicated that the distribution of both *Ae. aegypti* and *Ae albopictus* differed significantly among the studied districts at 95% level of confidence. The highest abundance of *Aedes* vectors was recorded from Kandy (46.95%) and Gampaha (41.28%) districts, which were among the high-risk areas recently for dengue within the country [19, 20].

### 3.2. Distance-Based Redundancy Analysis (dbRDA) for the Distribution Pattern of Mosquito Species

**Figure 2:**
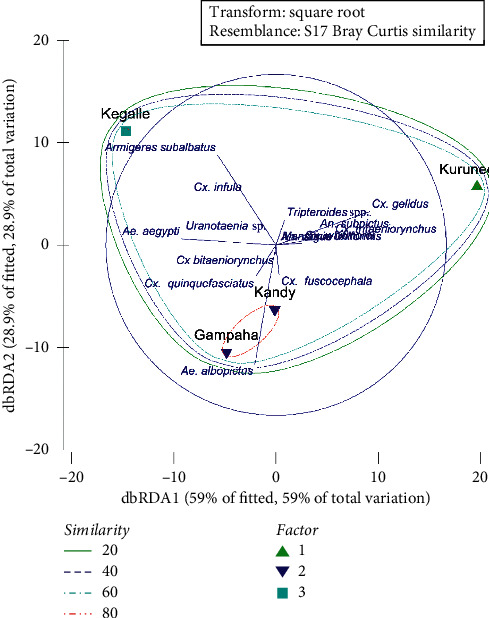
dbRDA plot for distribution of mosquito species in Kurunegala, Kegalle, Gampaha, and Kandy districts, Sri Lanka.

The relative distribution of different mosquito species differed significantly among the four districts (*X*^2^ = 143.248; *df* = 33; *P* < 0.001). Mosquito assemblages in Gampaha and Kandy districts indicated a similarity of 83.16%, while Kegalle district shared a similarity of 75.45% with the above cluster. On the other hand, Kurunegala shared only 69.99% similarity with the other two clusters, forming three subclusters based on the Bray–Curtis similarity (Figure 2). According to the loadings of the dbRDA axes, relatively higher densities of *An. subpictus, Cx. gelidus,* and *Cx. tritaeniorynchus* could be recognized as the reasons for the separation of Kurunegala district as a separate cluster, while a higher abundance of *Ae. aegypti* and *Armigeres subalbatus* could be recognized as the characteristic features of Kegalle district. The dominance of *Ae. albopictus* over *Ae*. *aegypti* and higher densities of *Cx. tritaeniorynchus* and *Tripteroides* sp. can be identified as the factors for the separation of Gampaha and Kandy districts as a single cluster (Figure 2).

### 3.3. Habitat Positivity

**Figure 3:**
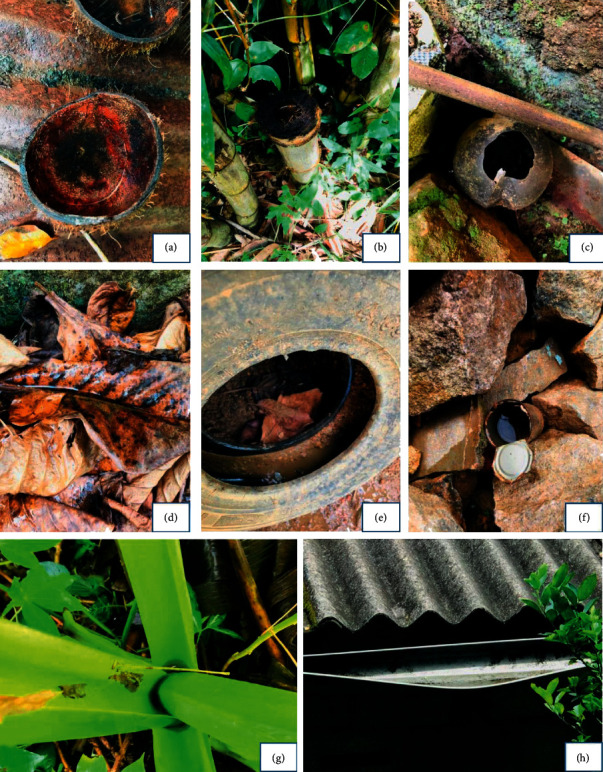
Immature mosquito habitats, (a) coconut shell, (b) bamboo tree hole, (c) clay pot, (d) leaf litter, (e) tire, (f) metal container, (g) leaf axil, and (h) roof gutter.

**Figure 4:**
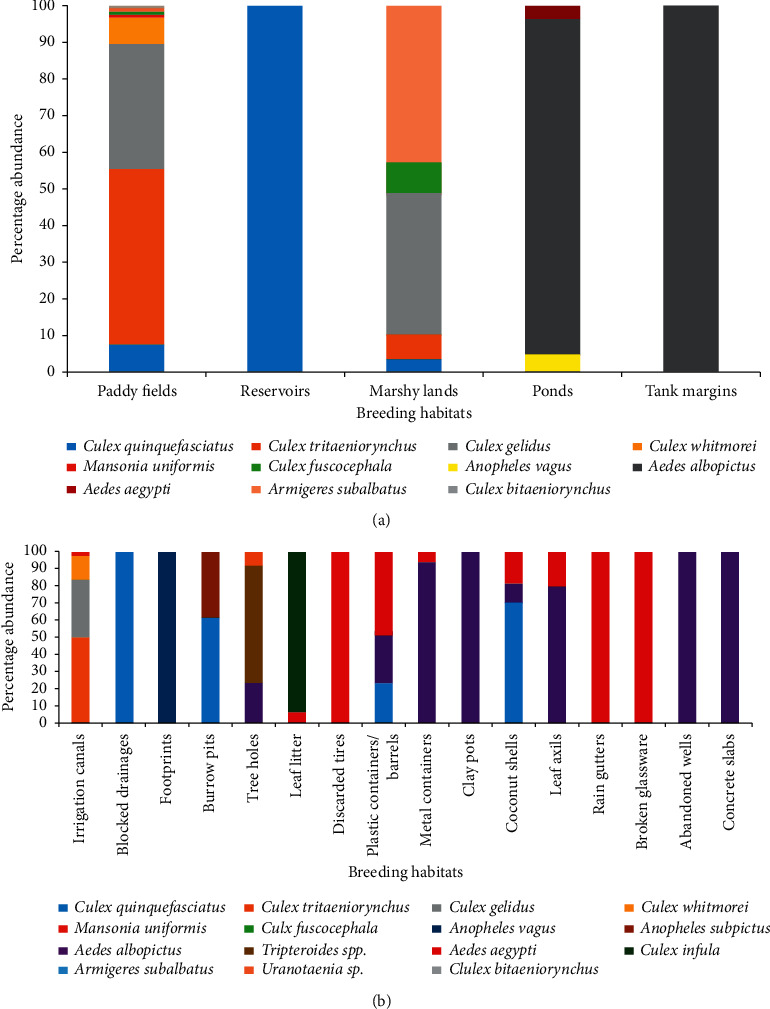
(a) The percentage abundance of mosquito species encountered in different macrobreeding habitats. (b) The percentage abundance of mosquito species encountered in different microbreeding habitats.

A total of 21 permanent/temporary key breeding sites were found from the study areas (Table 3). Paddy fields and Marshy lands were found in all four districts as permanent mosquito breeding habitats. The majority of the sampled breeding habitats belonged to the category of temporary microbreeding habitats (Figure 3). Five major macrobreeding habitat types, such as marshy lands, paddy fields, ponds, reservoirs, and tank margins, were positive for immature stages, while the highest larval diversity and abundance were found in paddy fields (*n* = 1399). The majority of species recorded from there belonged to the genus *Culex*. *Anopheles* larvae were only recorded from the tank margins and stagnant water bodies (Figure 4(a)). A higher proportion of temporary microbreeding habitats was occupied by *Ae. aegypti* and *Ae. albopictus* immature stages (Figure 4(b)), while *Ae. albopictus* showed a relatively higher distribution and abundance over *Ae. aegypti*.

Out of fifteen mosquito species recorded from the study area, ten species were medically important. Among them, *Ae. albopictus* exhibited the highest habitat diversity selection for breeding (Table 4). Majority of the breeding sites of *Ae. aegypti* and *Ae. albopictus* mosquitoes were temporary containers. Other than discarded container habitats, *Ae. aegypti* immature stages were found in water collected in fallen dried leaves, whereas *Ae. albopictus* larval stages were found from tree holes, stream margins, and abandoned and dried up wells and ponds. Only two species of *Anopheles* were recorded in the present study, and both species are medically important. About 73.3% of total *Cx. tritaeniorynchus* larvae collected were found in paddy fields.

### 3.4. Diversity of Mosquito Larvae within Breeding Habitats

As suggested by the Kruskal–Wallis statistics (*P* < 0.05 at 95% of significance), all the diversity indices for immature stages of medically important mosquitoes varied significantly across different breeding sites during the study (*k* = 20). Meanwhile, none of the diversity indices indicated significant differences in terms of locality (studied districts). Paddy fields had the highest species richness of 4.0 ± 2.82. Among all 21 different types of breeding sites recorded, the highest Shannon–Weiner diversity was observed from paddy fields (7.06 ± 2.82), while 14 breeding sites denoted a null Shannon–Weiner diversity, due to the presence of a single mosquito species (Table 5). The highest values of Pielou's index (2.01 ± 0.6), Menhinik's index (3.34 ± 0.83), and Margalef's index (0.59 ± 0.19) in terms of medically important mosquito larvae were also shown by paddy fields.

### 3.5. Interspecific Association of the Common Species

The coefficients of interspecific association among all the recorded medically important vector mosquitoes were negative. The lowest coefficient value remained as −0.05. *Ae. aegypti*, *Ar. subalbatus*, and *Mansonia uniformis* denoted the strongest negative coefficients of interspecific association (−0.13) for all the vector species. Therefore, different habitat preferences or interspecific repulsions among the reported vector stages are revealed by the negative associations between species.

## 4. Discussion

As a result of successive efforts in control programs, Sri Lanka has received remarkable achievements by receiving the WHO certification as malaria-free in 2016 and filariasis as no longer a public health concern [21]. However, despite all the successful efforts, some arbovirus infections (especially dengue) have increased rapidly over the last few decades. The current vector surveillance system is not systematized, and there is no regular monitoring of vector abundance. Some control programs only target the specified vectors for that disease, and reporting of other vector species is ignored. Knowledge of where mosquitoes breed, their habitat preference over others, and the larval distribution are important parameters for risk assessment and sound mosquito control strategies [22].

Water-holding containers are found to be the main larval breeding habitats for *Ae. aegypti* and *Ae. albopictus* in all the four districts. Only outdoor sampling of dengue vector mosquitoes was performed in the present study; hence, no information on the breeding of the vector indoors could be determined. Anyhow, Chan et al. [23] have stated that the majority of the habitats of *Ae. aegypti* larvae are also found indoors such as earthenware jars, tin cans, ant traps, rubber tires, bowls, and drums. The immature forms of *Ae. albopictus* were found in artificial containers with stagnant water besides natural habitats such as tree holes, rock holes, hollow bamboo stumps, and leaf axils [24], agreeing with findings of the current study. In Sri Lanka, dengue has become a significant socioeconomic and public health burden. *Aedes aegypti* is considered as the predominant vector, while *Ae. albopictus* is considered as a subsidiary vector of dengue in Sri Lanka. Although *Ae. aegypti* is considered as the primary or major vector of dengue [25], which initiate outbreaks and transmitting disease in highly urbanized areas, *Aedes albopictus* is also playing a significant role in transmitting dengue in suburban and rural areas [26].

Genus *Culex* indicated a diversified collection denoting seven species from all districts. Out of the seven reported species, three (*Cx. tritaeniorynchus, Cx, gelidus,* and *Cx. fuscocephala*) transmit Japanese encephalitis (JE) virus in Sri Lanka [27, 28]. Also, *Cx. quinquefasciatus*, which is a major vector for filariasis, was collected from all districts. Therefore, this signifies the entomological potential to transmit JE and filariasis infections in these districts. However, Sri Lanka intends to maintain the success of eliminating LF as a public health problem and prevent its resurgence. A few “hot spots” remain in the country, which necessitates continued vigilance. In Sri Lanka, LF was endemic in eight districts (Colombo, Kalutara, Gampaha, Galle, Matara, Hambantota, Kurunegala, and Puttalam), and about half of the population of Sri Lanka live in filariasis endemic districts [29]. Therefore, regular entomological monitoring activities, elimination of breeding sites, and continued special and routine surveillance activities are instrumental in achieving the elimination of filariasis.

Sri Lanka, being a country in which a considerable extent of land is occupied by irrigation reservoirs and paddy fields, those permanent habitats have become ideal breeding habitats for many mosquitoes. Major epidemics of JE have also occurred associated with the rice fields in Sri Lanka [30]. A study conducted in the Kelaniya area in Gampaha district of Sri Lanka has indicated the presence of four *Culex* mosquitoes namely *Cx. gelidus, Cx. tritaeniorynchus, Cx. fuscocephala,* and *Cx. pseudovishnui* from paddy fields [8]. Kurunegala district is an area with a higher abundance of rice fields and associated agricultural practices; thus, associated *Culex* mosquito abundance was found to be higher in those areas. Furthermore, rainfall patterns also may dilute the water in rice fields, altering their physicochemical properties, resulting in changes in larval density and species succession [31].

Kurunegala has been a malaria endemic district in Sri Lanka. Regardless of the few cases that have been reported from Kegalle and Kandy districts until the recent past, there were no indigenous cases identified from these districts, since 2012. The most recent indigenous cases have been identified from Kegalle, Kandy, Gampaha, and Kurunegala districts in 2009, 2010, 2010, and 2010, respectively [32–34]. The rapid influx of indigenous cases of malaria from foreign travelers has become a major challenge in the malaria prevention programs in Sri Lanka. In 2015, all 36 recorded cases of malaria were imported, with mortality being zero [35]. Classification of cases is based on information accruing from the rigorous case investigations, which are carried out on every case of malaria detected with a view of determining if there is a chance of the case being locally acquired. According to the available data at the Malaria campaign of Sri Lanka, during the last 2 years (2015-2016), 77 cases have been reported, the majority of which have been reported from the Western Province (Colombo, Gampaha, and Kalutara districts) [35, 36].

In the present investigation, only *An. subpictus* and *An. vagus* were identified from Kurunegala district, which was an early malaria endemic district in Sri Lanka. This may be due to the limited breeding habitats examined, and many of the breeding habitats investigated were temporary microbreeding habitats that may not be conducive for *Anopheles*. An ecological analysis of mosquitoes in rice fields conducted at Habaraluwewa area in Sri Lanka for 18 months revealed the presence of mosquitoes under genera *Anopheles*, *Aedes*, *Mansonia,* and *Armigeres*. Meanwhile, seepage pools and paddy fields were characterized with the highest density and occurrence of *Anopheles* and *Culex* species [28]. As well, six *Culex* species including *Cx. gelidus*, *Cx. tritaeniorynchus*, and *Cx. fuscocephala,* which are considered to be vectors for JE transmission in Sri Lanka [30, 37], were recorded from paddy fields and associated irrigation canals in the present study.

In the present study, the majority of *Cx. quinquefasciatus* was recorded from blocked drains. Muturi [38] has stated that *Cx. quinquefasciatus* prefers to breed in organically polluted water from the garbage-filled pools, ditches, and drains [39]. Unplanned urbanization in sampling areas had led to the increase of associated *Cx. quinquefasciatus* vector populations in those areas. From marshy lands, *Cx. gelidus* was sampled with a higher density. The presence of vegetation and floating plants provides optimal breeding conditions for *Cx. gelidus* by acting as food sources and shelter from predators [40], while vegetation creates stagnant conditions by decreasing water movements.

*Mansonia* species tend to lay eggs in vegetation-covered habitats, often on the under surfaces of floating leaves of aquatic plants [41]. Only *Mansonia uniformis* was recorded from the present investigation, which is considered to transmit brugian filariasis [42]. The current study highlights the necessity of integrated vector control strategies focusing on the different categories of breeding sites without limiting to a specific vector of interest in a control program with the co-occurrence of different medically important mosquitoes in the same breeding habitats.

Irregular disposal of household waste products such as polythene and artificial containers are major contributory factors for the increment of mosquito breeding places in the study area, also increasing the potential risk for disease transmission by enhancing the vector receptivity. Also, unplanned urbanization and town development in the study area created additional risk for disease transmission by increasing the number of mosquito breeding grounds. Mosquito replacement in an area could be occurred due to environmental changes caused by urbanization in the form of increased mosquito breeding habitats. This has been confirmed by a survey carried out in Colombo district, Sri Lanka [43]. Therefore, regular surveillance activities are of ample importance to identify the entomological potential for disease transmission with time, and this investigation emphasizes the necessity of an integrated program to survey medically important mosquitoes in all districts, which may ultimately be useful for successful elimination of vectors.

Furthermore, this would ultimately provide an avenue to improve the personal skills of health staff rather than limiting their knowledge to specified disease vectors under which the control program is concerned. On the other hand, this approach would be a beneficial solution to the limited trained staff and funding through the rational use of finances and human resources to achieve better productivity and fruitfulness of such programs. Therefore, this warrants vector control entities to rethink of a countrywide integrated surveillance and control approach for medically important disease vectors.

## 5. Conclusion

*Cx. gelidus* was the most predominant mosquito species in Kurunegala followed by *Cx. tritaeniorynchus*. *Ae albopictus* remained the most abundant mosquito species in Gampaha and Kegalle districts. Meanwhile, *Ae. aegypti* (19.19%) dominated in Kegalle district. The presence of medically important mosquitoes in these areas in considerable numbers can cause public health concerns as dengue is one of the major challenges in these areas. Therefore, the study of this nature would be useful to identify the entomological potential for disease transmission, and updates would be facilitated for implementing appropriate vector control interventions.

## Figures and Tables

**Table 1:** Ecological characteristics of the study areas.

Factor	District			
	Kurunegala	Gampaha	Kegalle	Kandy
Climatic zone	Intermediate	Wet	Wet	Wet
Average annual temperature (°C)	27.3 (rise up to 35°C in April)	27.3	26.3	24.5
Temperature range (°C)	25–32.5	25–32.5	25–30	17.5–25
Annual mean rainfall (mm)	1993	2398	2493	2083
Rainfall range (mm)	1000–2000	2000–3500	2000–3500	2000–6000

**Table 2:** The abundance of mosquito species in Kurunegala, Gampaha, Kegalle, and Kandy districts, Sri Lanka.

Genera	Species name	Kurunegala district		Gampaha district		Kegalle district		Kandy district	
		*n*	Relative abundance (%)	*n*	Relative abundance (%)	*n*	Relative abundance (%)	*n*	Relative abundance (%)
*Aedes*	*Ae. aegypti*	42	2.7	160	14.88	243	19.19	86	10.3
	*Ae. albopictus*	183	11.78	276	26.38	176	13.9	306	36.65

*Anopheles*	*An. subpictus*	110	7.08	0	0	0	0	0	0
	*An. vagus*	8	0.51	0	0	0	0	0	0

*Armigeres*	*Armigeres* sp.	0	0	0	0	140	11.06	0	0

*Culex*	*Cx. quinquefasciatus*	236	15.19	215	20.34	235	18.56	113	13.53
	*Cx. tritaeniorynchus*	394	25.35	180	16.87	197	15.56	161	19.28
	*Cx. gelidus*	411	26.45	117	10.61	134	10.58	112	13.41
	*Cx. whitmorei*	101	6.5	35	2.48	24	1.9	12	1.44
	*Cx. fuscocephala*	12	0.77	38	2.78	0	0	0	0
	*Cx. infula*	0	0	0	0	78	6.16	0	0
	*Cx bitaeniorynchus*	0	0	18	0.79	0	0	0	0

*Mansonia*	*Mansonia* sp.	10	0.64	22	1.19	0	0	0	0

*Tripteroides*	*Tripteroides* sp.	47	3.02	47	3.68	21	1.66	45	5.39

*Uranotaenia*	*Uranotaenia* sp.	0	0	0	0	18	1.42	0	0

Total mosquito larvae collected		1554		1108		1266		835	

**Table 3:** List of breeding habitats encountered in Kurunegala, Gampaha, Kegalle, and Kandy districts, Sri Lanka.

Nature of habitat	Breeding habitat (*n*)	District			
		Kurunegala	Gampaha	Kegalle	Kandy
*Macro*	Paddy field (23)	+	+	+	+
	Marshy land (12)	+	+	+	+
	Reservoir (2)	+	−	−	−
	Pond (6)	+	+	+	+
	Tank margin (4)	+	+	−	−

*Micro*	Irrigation canal (13)	+	+	+	+
	Blocked drain (10)	+	+	+	+
	Tree hole (16)	+	+	+	+
	Plastic container/barrel (13)	+	+	+	+
	Burrow pit (4)	+	+	−	−
	Tire (6)	+	+	+	+
	Leaf axil (5)	−	+	+	+
	Rain gutter (3)	−	−	+	+
	Metal container (15)	+	+	+	+
	Glassware (3)	−	−	+	+
	Coconut shell (7)	−	+	+	+
	Leaf litter (5)	+	−	+	+
	Clay pot (2)	+	−	−	−
	Footprint (3)	+	+	−	−
	Abandoned well (4)	−	+	+	+
	Concrete slab (4)	−	+	+	+

Note: number of habitats sampled included in parentheses.

**Table 4:** The abundance of the immature stages of medically important mosquito species encountered in each breeding habitat.

Genera	Species	Habitat type	Breeding habitat	Relative abundance	Immature stages (*n*)	Relative abundance (%)
*Aedes*	*Ae. aegypti*	Macro	Pond	6	6	0.14
		Micro	Plastic container/barrel	41	290	6.74
			Leaf litter	2	5	0.12
			Metal container	4	22	0.51
			Roof gutter	24	71	1.65
			Glassware	38	38	0.88
			Leaf axil	3	3	0.07
			Coconut shell	13	26	0.60
			Tire	9	56	1.30
	*Ae. albopictus*	Macro	Tank margin	13	40	0.93
			Pond	30	152	3.53
		Micro	Plastic container/barrel	41	163	3.79
			Clay pot	18	36	0.84
			Concrete tube	17	68	1.58
			Tree hole	10	51	1.19
			Glassware	21	41	0.95
			Leaf axil	3	12	0.28
			Coconut shell	7	29	0.67
			Abandoned well	4	14	0.33
			Metal container	37	329	7.65

*Anopheles*	*An. subpictus*	Micro	Burrow pit	110	110	2.56
	*An. vagus*	Macro	Stream margin	8	8	0.19

*Armigeres*	*Armigeres* sp.	Macro	Marshy land	47	140	3.25

*Culex*	*Cx. quinquefasciatus*	Macro	Marshy land	12	12	0.28
			Reservoir	42	84	1.95
			Paddy field		108	2.51
		Micro	Blocked drain	19	185	4.30
			Plastic container	68	135	3.14
			Coconut shell	26	26	0.60
			Burrow pit	58	173	4.02
	*Cx. tritaeniorynchus*	Macro	Paddy field	56	676	15.71
			Marshy land	11	22	0.51
		Micro	Irrigation canal	41	224	5.21
	*Cx. gelidus*	Macro	Paddy field	69	485	11.27
			Marshy land	21	127	2.95
		Micro	Irrigation canal	38	152	3.53
	*Cx. whitmorei*	Macro	Paddy field	25	100	2.32
		Micro	Irrigation canal	21	62	1.44

*Mansonia*	*Mansonia* spp.	Macro	Paddy field	10	10	0.23
		Micro	Irrigation canal	6	12	0.28

**Table 5:** Diversity of medically important mosquito species encountered in different breeding habitats across the four districts.

Breeding site	Species richness	Shannon–Weiner index	Menhinik's index	Margalef's index	Pielou's index	Dominance
Paddy field	7^a^	7.06 ± 2.8^a^	3.34 ± 0.83^a^	0.59 ± 0.19^a^	2.01 ± 0.6^a^	0.28 ± 0.06^a^
Reservoir	1^d^	0	0.03 ± 0.01^d^	0	0	0.01 ± 0.00^c^
Tank margin	1^d^	0.57 ± 0.4^c^	0.11 ± 0.04^d^	0.32 ± 0.12^b^	0.21 ± 0.1^c^	0.01 ± 0.00^c^
Marshy land	5^b^	1.52 ± 0.5^b^	1.50 ± 0.26^b^	0.32 ± 0.09^b^	0.52 ± 0.2^b^	0.06 ± 0.02^b^
Pond	3^c^	0	0.10 ± 0.03^d^	0.06 ± 0.02^d^	0	0.04 ± 0.01^c^
Irrigation canal	4^b^	1.86 ± 0.4^b^	0.99 ± 0.21^b^	0.36 ± 0.11^b^	0.59 ± 0.2^b^	0.09 ± 0.03^b^
Blocked drainage	1^d^	0	0.04 ± 0.01^d^	0.11 ± 0.04^d^	0	0.06 ± 0.02^b^
Burrow pit	2^c^	0	0.02 ± 0.01^d^	0.02 ± 0.01^d^	0	0.06 ± 0.01^b^
Tree hole	3^c^	1.56 ± 0.3^b^	0.83 ± 0.26^c^	0.38 ± 0.09^b^	0.59 ± 0.1^b^	0.05 ± 0.01^c^
Leaf litter	2^c^	0	0.11 ± 0.03^d^	0.03 ± 0.01^d^	0	0.02 ± 0.01^c^
Tire	1^d^	0	0.11 ± 0.02^d^	0.10 ± 0.04^d^	0	0.01 ± 0.00^c^
Plastic container	3^c^	1.56 ± 0.6^b^	1.02 ± 0.32^b^	0.13 ± 0.05^d^	0.48 ± 0.1^b^	0.12 ± 0.04^b^
Metal container	2^c^	0	0.03 ± 0.01^d^	0.11 ± 0.06^d^	0	0.09 ± 0.03^b^
Clay pot	1^d^	0	0.04 ± 0.01^d^	0	0	0.01 ± 0.00^c^
Footprint	1^d^	0	0.12 ± 0.02^d^	0	0	0
Roof gutter	1^d^	0	0	0.09 ± 0.03^d^	0	0.02 ± 0.00^c^
Glassware	1^d^	0	0	0.08 ± 0.02^d^	0	0.02 ± 0.00^c^
Leaf axil	2^c^	0	0	0.22 ± 0.09^c^	0	0
Coconut shell	3^c^	1.23 ± 0.8^b^	0.55 ± 0.11^c^	0.20 ± 0.09^c^	0.47 ± 0.2^b^	0.02 ± 0.00^c^
Concrete slab	1^d^	0	0	0.09 ± 0.03^d^	0	0.02 ± 0.01^c^
Abandoned well	1^d^	0	0	0.07 ± 0.02^d^	0	0

Note: different superscript letters in each column (a, b, c, and d) denote significant variations suggested by the Kruskal–Wallis statistics at 95% of significance, followed by the Dunn test with Bonferroni adjustment as the post hoc test.

## Data Availability

The datasets supporting this study are included within the article. Data will not be shared in any of the sources.

## Supplementary Materials

Supplementary Materials Annexure 1a: percentage abundance of different mosquito species within the Gampaha district. Annexure 1b: percentage abundance of different mosquito species within the Kandy district. Annexure 1c: percentage abundance of different mosquito species within the Kegalle district. Annexure 1d: percentage abundance of different mosquito species within the Kurunegala district.
