# Supplementary material for: Breeding Habitat Distribution of Medically Important Mosquitoes in Kurunegala, Gampaha, Kegalle, and Kandy Districts of Sri Lanka and Potential Risk for Disease Transmission: A Cross-Sectional Study
Source: J Trop Med. 2020 Sep 2;2020:7915035. doi: 10.1155/2020/7915035 (PMC7484687; doi:10.1155/2020/7915035)
Supplement: Supplementary Materials — Annexure 1a: percentage abundance of different mosquito species within the Gampaha district. Annexure 1b: percentage abundance of different mosquito species within the Kandy district. Annexure 1c: percentage abundance of different mosquito species within the Kegalle district. Annexure 1d: percentage abundance of different mosquito species within the Kurunegala district. [file 7915035.f1.docx]

**Annexure 1**


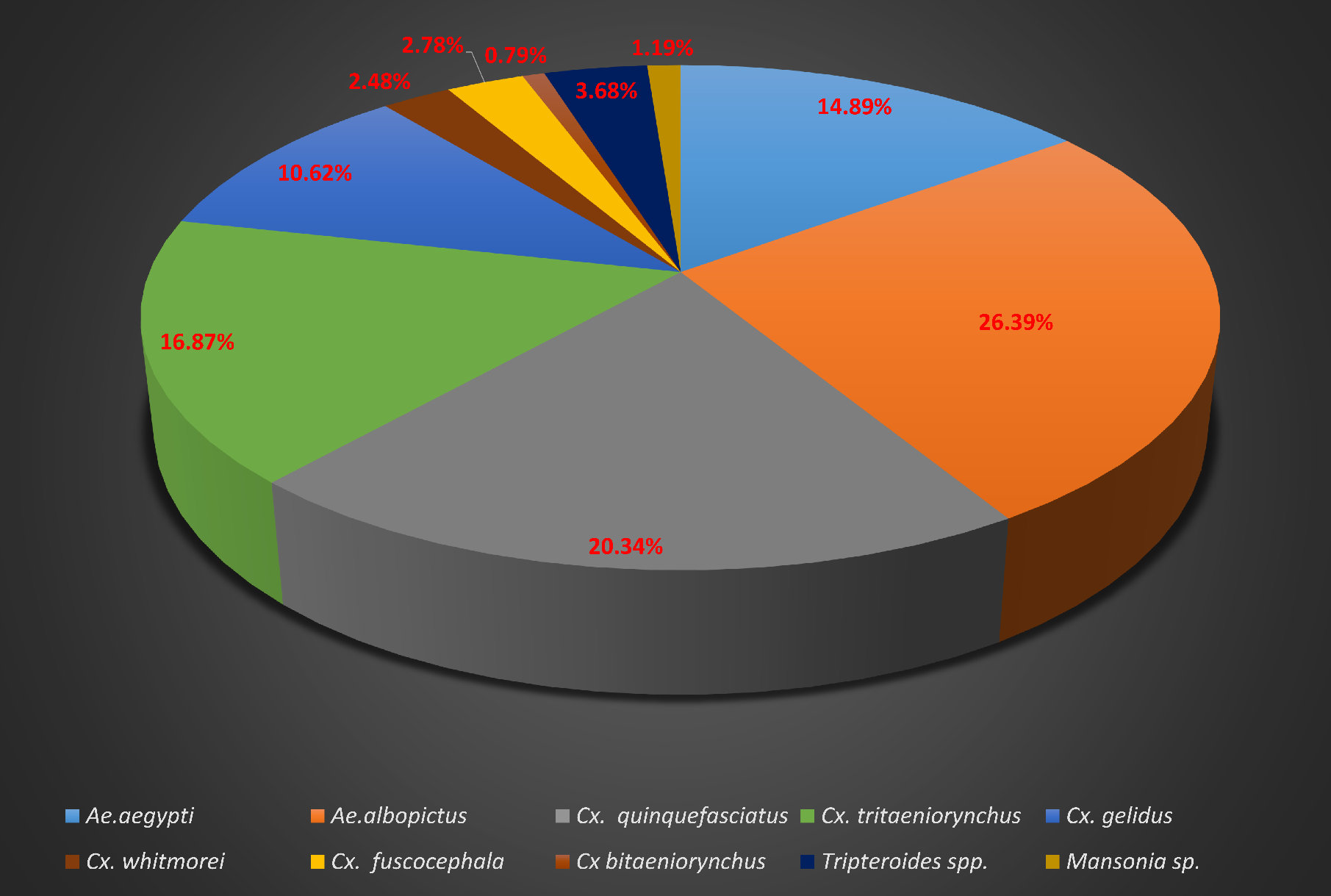


**Annexure 1a.** Percentage abundance of different mosquito species within the Gampaha district


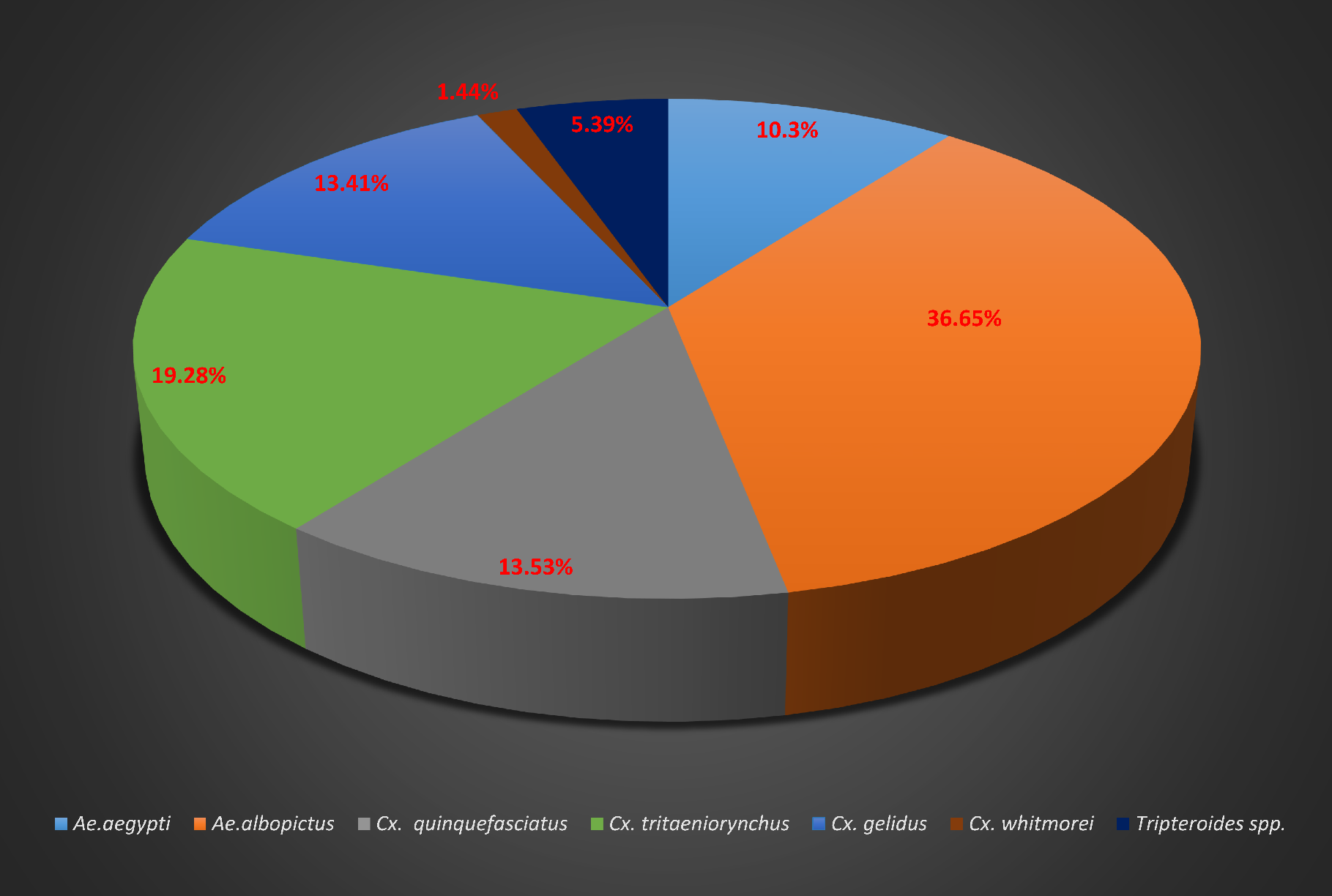


**Annexure 1b.** Percentage abundance of different mosquito species within the Kandy district


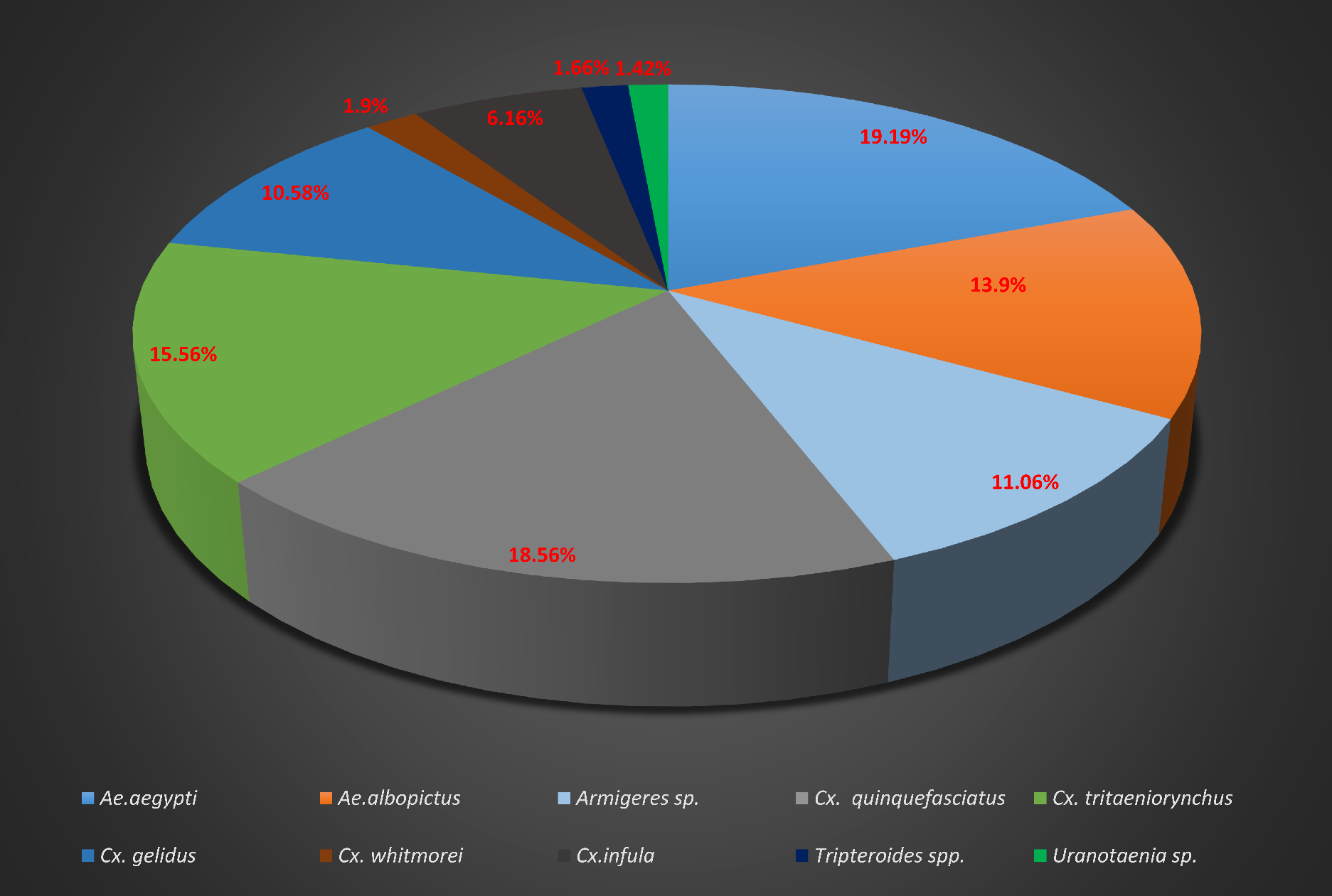


**Annexure 1c.** Percentage abundance of different mosquito species within the Kegalle district


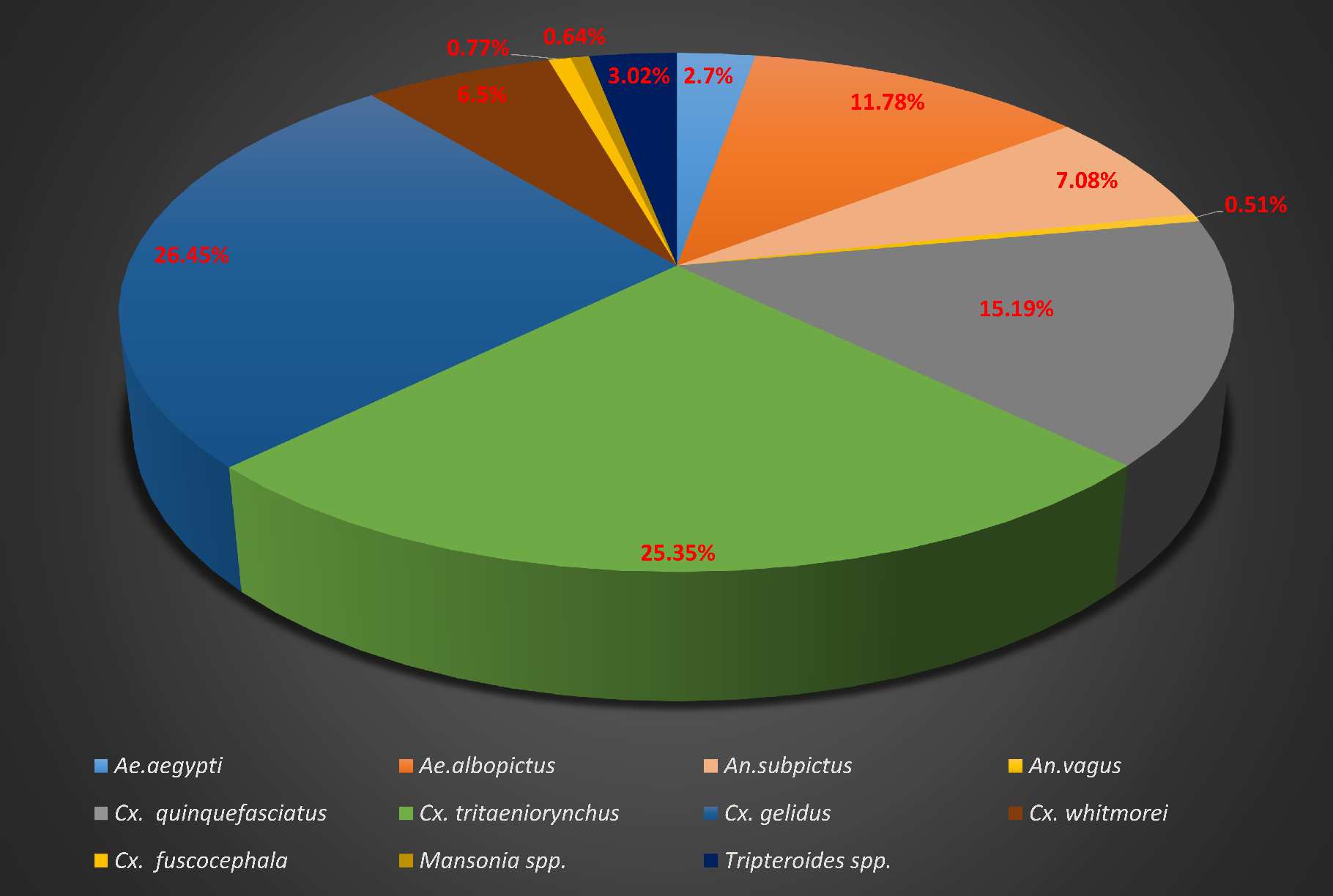


**Annexure 1d.** Percentage abundance of different mosquito species within the Kurunegala district
